# Editorial: Advancing therapeutic strategies: exploring ABC transporters and chemicals affecting their expression and function for disease treatment

**DOI:** 10.3389/fphar.2024.1423979

**Published:** 2024-05-13

**Authors:** Guido Veit, Michinori Matsuo, Tsukasa Okiyoneda

**Affiliations:** ^1^ Department of Physiology, McGill University, Montréal, QC, Canada; ^2^ Department of Food and Nutrition, Faculty of Home Economics, Kyoto Women’s University, Kyoto, Japan; ^3^ Department of Biomedical Sciences, School of Biological and Environmental Sciences, Kwansei Gakuin University, Sanda, Japan

**Keywords:** ABC transporters, CFTR (cystic fibrosis transmembrane conductance regulator), pharmacological chaperones, missense mutations, premature termination codon (PTC)

## Introduction

ATP-binding cassette (ABC) transporters are ubiquitously expressed multipass membrane proteins that transport ligands across biological membranes, providing multiple critical functions for cellular and organismal physiology. In humans, 48 members of this superfamily are present, of which at least 21 are linked to rare monogenetic disorders, and many are implicated in complex diseases (Moore et al., 2023). These monogenetic disorders result from loss-of-function caused by nonsense or frameshift causing mutations that lead to loss of protein expression, or missense mutations that either cause folding/processing defects or defects in protein function (Dean et al., 2022; Moore et al., 2023).

For the cystic fibrosis transmembrane conductance regulator (CFTR/ABCC7), the more than 2,100 identified mutants have been classified according to their molecular cell biological and functional phenotypes into six distinct classes: Class I—protein synthesis defect, Class II—maturation defect, Class III—gating defect, Class IV—conductance defect, Class V—reduced quantity, and Class VI—reduced PM stability, with many mutants exhibiting characteristics of more than one class (Veit et al., 2016). CFTR is so far the only ABC transporter targeted by approved modulator drugs that either promote the folding (correctors) or the function (potentiators) of cystic fibrosis (CF)-causing mutants carrying missense mutations. The most efficacious of these modulator drugs, Trikafta, containing the folding corrector tezacaftor, the gating potentiator ivacaftor, and the dual-acting corrector and potentiator elexacaftor, provides unprecedented clinical benefit to patients carrying the most common CF causing mutation F508del and has also been FDA-approved for >170 rare missense mutants (Heijerman et al., 2019; Middleton et al., 2019; Lopes-Pacheco et al., 2021; Veit et al., 2021). The physiological effects of Trikafta therapy are so far incompletely understood, and some rare missense mutants attain no or insufficient correction, thus requiring further modulator development. For CFTR nonsense mutants, predominantly owed to their complex molecular therapeutic phenotypes, modulator therapy is not yet available.

It is hoped that similar strategies as for the development of CFTR modulators can be employed to isolate gain-of-function modulators for other ABC transporters. This is exemplified by the responsiveness of a variety of mutant ABC transporters to approved CFTR modulator drugs and 4-phenylbutyrate in cell assays (Vauthier et al., 2017). A prerequisite for the wide adoption of such approaches, however, is a better understanding of the structure-function relationship of ABC transporters.

## Improving targeted CFTR modulator therapy and understanding the physiological modulator effects

In this special Research Topic we collected four studies that use different perspectives on advancing therapeutic strategies for ABC transporters. Three of these studies explore different aspects of modulator therapy for CF.

Zajac et al. reported that Trikafta rescues the defective HCO_3_^−^ secretion in F508del homozygous CF airway epithelia to a level corresponding to 80% of that of wild-type (WT) epithelia, which, however did not result in an increase in the airway-surface liquid pH to the WT level. In contrast, treatment with the pro-inflammatory cytokines TNFα and IL-17 dramatically increased the mRNA expression of the Cl^−^/HCO_3_^−^ exchanger SLC26A4 as well as normalized HCO_3_^−^-secretion and ASL pH, which was further augmented by Trikafta. These results therefore provide further insights into the mechanism that underlies increased CFTR modulator efficacy in the presence of inflammation (Gentzsch et al., 2021; Rehman et al., 2021).

Taniguchi et al. isolated novel corrector molecules that are additive to Trikafta for the correction of F508del and rare CFTR missense mutants. The authors used a training set of known CFTR modulators and a machine-learning model to virtually screen over 4 million compounds. This approach identified one compound (FR3) with a distinct mechanism to approved modulators, stabilizing the nucleotide-binding domain 1 of CFTR. FR3 also corrected the defective plasma membrane expression of a misfolded mutant ABCB1, thus providing further credence to the idea that modulators can target multiple ABC transporters. FR3 may also show efficacy for ABCA3 mutants with established responsiveness to CFTR correctors (Kinting et al., 2018). Therefore, evaluating the FR3 effect on various misfolded ABC transporters could prove valuable.

To study a subset of nonsense mutations, Premchandar et al. developed an affinity purification tandem mass spectrometry pipeline that allows the selective isolation of full-length CFTR molecules to determine the read-through efficacy and to determine the relative incorporation percentages of near-cognate amino acids at the premature termination codons. The authors observed different amino acid misincorporation ratios compared to those found in short reporter constructs, suggesting that the transcript sequence beyond the proximity of PTCs can impact the amino acid incorporation. The consequence of misincorporated amino acids on the CFTR folding and function was investigated and aided in optimizing CFTR modulator combinations. The study, therefore, provides a basis for refined mutation-dependent therapeutic strategies for various CF-causing nonsense mutations.

## Applying CFTR research strategies to other ABC-transporters

In their study Cui et al. set up a Baculovirus/Sf9 heterologous expression system to study the ATPase activity of human CFTR (hCFTR), lamprey CFTR (lp-CFTR) - the oldest known CFTR ortholog, and human ABCC4 (hABCC4) - the closest mammalian paralog to CFTR, in the same lipid environment condition. Using the sensitive antimony-phosphomolybdate assay, the authors showed that the ATPase activity of hCFTR is significantly higher than that of lp-CFTR, and that the intrinsic ATPase activity of hABCC4 tested in the absence of substrates is about twice of that of hCFTR. This study supports the notion that the lipid environment modulates the activity of ABC transporters, and contributes to understanding the structure-function relationship of ABC transporters.

We expect that this Research Topic will contribute to the foundation necessary to develop new therapeutic strategies for diseases associated with the dysregulation of ABC transporters.
